# Drowning at Sea

**Published:** 1878-11

**Authors:** 


Drowning at Sea.—A person who will throw himself on his back in the water, with his hands held clasped in each other at his back, and with his head thrown back so that the nose and mouth may protrude from the water, may float for hours and cannot sink in that position.

A common feather pillow tied around under the arms is said to be worth half-a-dozen common India rubber life preservers, while a common mattrass placed on a blanket, a trunk on the mattrass, then both trunk and mattrass tied up in the blanket and all thrown into the water together, will float with the tide for many hours.

---

The physicians of the New York Hospital give some facts, showing that seven-eights of all persons attacked by Cholera are those who have already been long suffering from some organic disease, as of the liver, lungs, &c., and who could not live long under any circumstances.

---

Lord Shaftesbury says that he would be virtuous for his own sake, though nobody were to know it; as he would be clean for his own sake, though nobody were to see him.

